# If started early in life, metformin treatment increases life span and postpones tumors in female SHR mice

**DOI:** 10.18632/aging.100273

**Published:** 2011-02-21

**Authors:** Vladimir N. Anisimov, Lev M. Berstein, Irina G. Popovich, Mark A. Zabezhinski, Peter A. Egormin, Tatiana S. Piskunova, Anna V. Semenchenko, Margarita L. Tyndyk, Maria N. Yurova, Irina G. Kovalenko, Tatiana E. Poroshina

**Affiliations:** N.N.Petrov Research Institute of Oncology, Pesochny-2, St.Petersburg 197758, Russia

**Keywords:** Metformin, age of initiation, biomarkers of aging, life extension, spontaneous carcinogenesis, mice

## Abstract

Hyperglycemia and hyperinsulinemia accelerate both aging and cancer. Antidiabetic biguanides such as metformin decrease glucose, insulin and IGF-1 level. Metformin increases lifespan and prevents cancer in mice, although its effects vary, depending on mice strain and gender. Here we showed that chronic treatment of female outbred SHR mice with metformin started at the age of 3, 9 or 15 months decreased body temperature and postponed age-related switch-off of estrous function. Surprisingly, metformin did not affect levels of serum cholesterol, triglycerides, glucose and insulin. Treatment with metformin started at the age of 3 months increased mean life span by 14% and maximum life span by 1 month. The treatment started at the age of 9 months insignificantly increased mean life span by only 6%, whereas the treatment started at the age of 15 months failed to increase life span. The mean life span of tumor-free mice was increased by 21% in ‘the youngest group’, by 7% in ‘middle-aged group’ and in contrast was reduced by 13% in ‘the oldest group’. When started at the age of 3 and 9 months, metformin delayed the first tumor detection by 22% and 25%, correspondingly. Thus, in female SHR mice, metformin increased life span and postponed tumors when started at the young and middle but not at the old age. In contrast, metformin improves reproductive function when started at any age.

## INTRODUCTION

The potential link between aging and insulin/IGF-1 signaling has attracted substantial attention during last years. This connection was evidenced by an increase in incidence of insulin resistance and type 2 diabetes in accelerated aging syndromes in humans, on the one hand, as well as by life span extension due to caloric restriction (CR) in rodents, on the other hand. Concomitant reduction in plasma insulin and plasma glucose levels, which implies increased sensitivity to insulin, emerges as a hallmark of increased longevity [1]. Hyperglycemia is an important aging factor involved in generation of advanced glycosylation end products (AGEs) [2]. There is evidence that hyperinsulinemia favors accumulation of oxidized proteins [2]. Untreated diabetics with elevated glucose levels suffer many manifestations of accelerated aging, such as impaired wound healing, obesity, cataracts, vascular and microvascular damage [3]. It is important to stress that hyperinsulinemia is a significant factor not only in aging but also in the development of cancer [3-5].

The concept of CR mimetics is now being intensively explored [6,7]. CR mimetics involve interventions that produce physiological and anti-aging effects similar to CR. It was suggested to use antidiabetic biguanides as a potential anti-aging treatment [3,10-14]. The antidiabetic drugs, phenformin and buformin, were observed to reduce hyperglycemia and produce the following effects: improved glucose utilization, reduced free fatty acid utilization, gluconeogenesis, serum lipids, insulin and IGF-1, and reduced body weight both in humans (including cancer patients) and experimental animals [3,15-17]. There are evidences of geroprotective and anticarcinogenic potential of metformin [11,17-21]. Of note, in all experiments related to the study of potential geroprotective action the treatment with metformin was started at the young age. In the same time, the possibility of a successful treatment with geroprotectors started at the middle or old age is very appealing [22]. There are data which ascribe some distinctions effects in regard of the caloric restriction or other geroprotective agents depending on the stage of ontogenesis when the exposure has been started and to the duration of the latter [22,23].

In this paper we present the results of treatments with the antidiabetic biguanide metformin which were started at the age of 3, 9 or 15 months in female outbred SHR mice.

## RESULTS

### Age-related body weight dynamics

**Figure 1.**
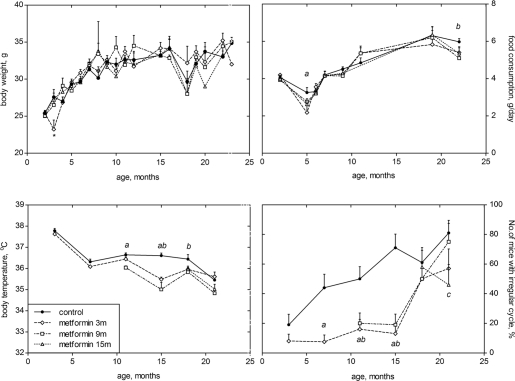
Age-related dynamics of body weight (**A**), food consumption (**B**), body temperature (**C)** and of the incidence of mice with irregular estrous cycles (**D**) in SHR mice not treated and treated with metformin starting at the age of 3, 9 or 15 months. The difference with the controls is significant, p<0.05: а- metformin 3 m; b - metformin 9 m; c - metformin 15 m)

The body weight of mice in both control and metformin-treated groups increased with age, exceeding by 16 months the body weight of 3-month-old animals by 34.31%. There was no difference in the mean body weight of mice exposed and non-exposed to the drug until the age of 20 months, and a tendency to a decrease of the body weight was observed in metformin treated groups after this age (Fig. 1A).

### Age-related dynamics of food and water consumption

The amount of food consumed daily by mice during the period of observation was similar in the control group and in metformin-treated groups (Fig. 1B). The amount of water consumed by mice varied during the period of observation and was practically the same in all groups (data are not shown).

### Age-related dynamics of estrous function in mice

The length of estrous cycles in the control female SHR mice was not significantly changed with age, whereas it was significantly decreased at various age in mice exposed to metformin from the age of 3, 9 or 15 months in comparison with the estrous cycle status in controls of corresponding age (Table 1).The fraction of mice with irregular estrous cycles increased with age in thecontrol mice but not in mice treated with metformin until the age of 15 months (Table 1, Fig. 1D), independently on the time of the start of metformin treatment.

**Table 1.** Effect of metformin given at the various age on age-related dynamics of estrous functional parameters in SHR mice.

Age, months	No. of mice		Length of estrous cycle, days	Rate of estrous cycles of various length, %			Fraction of mice with regular cycles, %
				< 5 days	5-7 days	> 7 days	
***Control***							
3		30	6.9 ± 0.28	0	64	36	81
7		29	8.1 ± 0.46	0	44	56	56
11		36	7.5 ± 0.50	0	44	56	50
14-15		24	7.4 ± 0.56	0	71	29	29
18		23	8.1 ± 0.56	0	33	67	39
21		21	8.0 ± 0.49	0	25	75	19
***Metformin, 3 months***							
3		33	7.1 ± 0.26	0	64	36	92
7		32	6.5 ± 0.24 **	3	74 *	23 *	92,5 ***
11		30	6.2 ± 0.31 *	27	50	23 *	84 **
14-15		24	6.4 ± 0.34	3,7	81,5	14,8	87 **
18		20	7.7 ± 0.52	0	40	60	50
21		14	6.5 ± 0.37 *	0	75	25	43
***Metformin, 9 months***							
11		32	6.1 ± 0.30 *	22	56	22 *	80 **
14-15		29	6.6 ± 0.32	7	76	17	81**
18		22	7.7 ± 0.41	0	50	50	50
21		12	7.7 ± 1.37	0	67	33	25
***Metformin, 15 months***							
18		19	7.6 ± 0.37	0	50	50	42
21		13	6.4 ± 0.50 *	0	87 *	13 *	54*

The difference with the controls of corresponding age is significant: * p < 0.05; ** p < 0.01; *** p < 0.001.

### Age-related dynamics of body temperature in mice

There was no significant age-related changes in average body temperature in the control mice. Treatment with metformin started at the age of 3 months decreased body temperature at the age of 15 months as compared with age-matched controls. Also, body temperature was decreased between the 11th and 18th months of life in mice treated with the drug from the age 9 months. Metformin administration started at the age of 15 months failed influence the average body temperature in mice (Fig. 1C).

### Effect of metformin on metabolic and hormonal parameters in mice

Treatment with metformin started at the age of 3, 9 or 15 months failed influence levels of glucose, total cholesterol, triglycerides, and insulin in the serum estimated when mice were 16.5 month old (Table 2).

**Table 2.** Effect of metformin on metabolic parameters in 16.5-month-old female mice

Treatment	Age at the start of metformin treatment	Glucose, mmol/l	Total cholesterol, mmol/l	Triglycerides, mmol/l	Insulin, μUnits/ml
Control	-	7.3 ± 1.50	2.9 ± 1.06	1.5 ± 0.46	0.17 ± 0.12
Metformin	3 months	8.1 ± 0.64	2.7 ± 0.37	1.0 ± 0.03	0.49 ± 0.47
	9 months	7.9 ± 0.59	2.5 ± 0.27	0.9 ± 0.22	0.06 ± 0.05
	15 months	7.8 ± 0.78	2.6 ± 0.21	1.1 ± 0.08	0.19 ± 0.18

### Survival and longevity of female SHR mice

**Figure 2.**
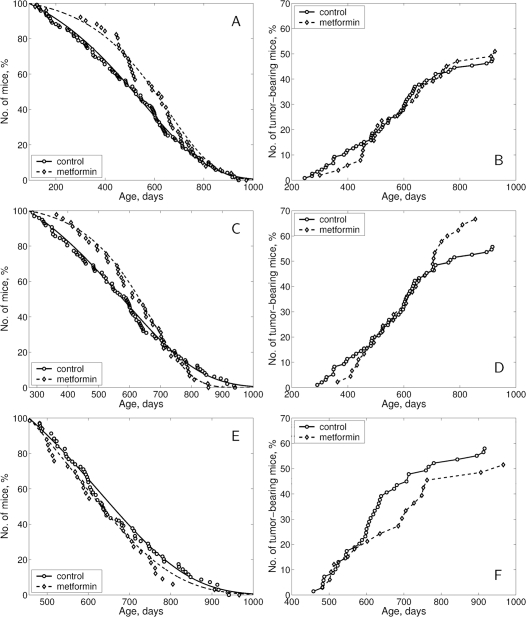
Survival curves and tumor yield curves in female in SHR mice not treated and treated with metformin starting at the age of 3 (**A, B**), 9 (**C, D**) or 15 months (**E, F**).

Treatment with metformin started at the age of 3 months shifted to the right the survival curve as compared with the controls (Fig. 2A). The effect of the treatment started at the age of 9 months was less expressed (Fig. 2C) and it was absent in the group with metformin started at oldest age (Fig. 2E). According to the log-rank test the difference in survival of female SHR mice subjected to metformin treatment from the age of 3, 9 or 15 months, compared to the age-matched control groups, is insignificant (p-value is 0.181; 0.97 and 0.478, correspondingly). According to the estimated parameters of the Cox's regression, metformin treatment started at the age of 3 months decreased the relative risk of death compared to the control group (β = −0.227; exp (β = 0.797; se (β)= 0.169; p = 0.173). Metformin treatment started at the age of 9 or 15 months produced no effect on the relative risk of death in SHR female mice compared to the control group.

The differences in survival were reflected in parameters of longevity. Metformin treatment started at the age of 3 or 9 months increased mean life span (+14.1% and 6.1%, correspondingly, p>0.05), median by 17.5% and 6.6%, correspondingly, but practically did not influence when started at the age of 15 months. Maximum life span was increased by 1 month only in mice treated from youngest age (Table 3). It is worthy to note that the mean life span of tumor-free mice has increased by 20.7% in ‘the youngest age started’ group, by +7.1% in ‘middle-age started’ group and was reduced by 12.8% in the ‘old age started’ group. Parameter α of the Gompertz model was higher by 1.4 times in the group subjected to metformin treatment from the age of 3 months and by 1.9 times in the middle-age started group as compared with the controls (p < 0.05). At the same time, it was slightly reduced in the old age started group treated with metformin. The difference between the control and this latter group was statistically significant (Table 3).

**Table 3.** Effect of metformin given from various age on life span in female SHR mice

Parameters	Control-3	MF-3	Control-9	MF-9	Control-15	MF-15
No. of mice at start of the treatment	119	51	97	45	69	33
Mean life span, days	511 ± 20.3	583 ± 26.7 (+14.1%)	583 ± 17.9	619 ± 19.5 (+6.2%)	668 ± 15.7	647 ± 21.4
Median, days	525	617	591	630	636	636
Mean life span of last 10%, days	881 ± 12.8	897 ± 27.8	892 ± 12.4	820 ± 14.1	913 ± 8.8	892 ± 47.0
Maximum life span, days	941	972	941	855	941	966
α × 10^3^, days^−1^	3.7 (3.2; 3.9)	5.2* (4.9; 5.3)	4.1 (3.9; 4.2)	7.8* (7.7; 8.2)	4.7 (4.6; 4.8)	4.3* (4.2; 4.5)
MRDT, days	187 (178; 217)	133* (131; 141)	169 (165; 178)	89* (85; 90)	147 (144; 151)	161* (154; 165)
No. of tumor-free mice	62	25	45	15	29	16
Mean life span of tumor-free mice, days	478 ± 32.7	577 ± 44.3 (+20.7%)	609 ± 29.7	652 ± 31.5 (+7.1%)	719 ± 23.9	627 ± 25.5* (−12.8%)

MRDT, mortality rate doubling time; in brackets - 95% confidential interval.

* The difference with the relevant controls is significant, p < 0.05.

### Development of spontaneous tumors in female SHR mice

According to the long-rank test there were no significant differences in age-related distributions of the total tumors occurrence in control and metformin-treated groups (Fig. 2). The first tumor-bearing mice (malignant lymphoma) died at the age of 246 days in the control group and at the 300th day (+22%) in the group of mice treated with metformin starting at 3 mo, while at the 363rd day (+24.7%) in the group exposed to metformin starting from middle-age and at 482nd day (+5.2%) in the oldest metformin-treated group as compared to the age-matched controls (Table 4). The mean life spans of tumor-bearing mice were similar being increased by 7.9%, 7.3% and 5.4% in groups treated with metformin from the age 3, 9 and 15 months respectively.

**Table 4.** Effect of metformin given from various age on spontaneous tumor development in female SHR mice

Parameters	Control-3	MF-3	Control-9	MF-9	Control-15	MF-15
Effective number of mice^a^	103	48	97	45	69	33
Age of the death of the 1^st^ TBM	246	300	291	363	458	482
Number of TBM^b^ (%)	57 (55.3%)	26 (54.2%)	54 (55.7%)	30 (66.7%)	40 (58.0%)	17 (51.5%)
Total number of tumors	60	26	57	31	43	18
Number of tumors per TBM	1.05	1.00	1.06	1.03	1.08	1.06
Number of malignant TBM (%)	54 (49.5%)	26 (54.2%)	51 (52.6%)	27 (60.0%)	37 (53.6%)	16 (48.5%)
Mean life span of TBM, days	547 ± 22.2	590 ± 31.3 (+7.9%)	562 ± 21.5	603 ± 24.4 (+7.3%)	631 ± 18.9	665 ± 34.1 (+5.4%)
*Localization and type of tumors*						
Mammary adenocarcinoma	42 (40.8%)	23 (47.9%)	40 (41.2%)	20 (44.4%)	27 (39.1%)	12 (36.4%)
Leukemia/lymphoma	7 (6.8%)	2 (4.2%)	6 (6.2%)	3 (6.7%)	6 (8.7%)	3 (9.1%)
Ovary: hemangioma granulesa-cell tumor adenocarcinoma	1 1 1	- - 1	1 1 1	- - 2	1 1 1	- - -
Liver: holangiocarcinoma	2	-	2	-	2	-
Lung: adenoma adenocarcinoma	1 3	- -	1 3	2 2	1 3	1 -
Skin: paplilloma fibrosarcoma	1 -	- -	1 -	1 -	1 -	- 1
Soft tissues: sarcoma	-	-	-	-	-	1
Utery: hemangioma	-	-	-	1	-	-
Spleen: hemangioma	1	-	1	-	1	-

a, number of mice surviving the first tumor bearing animal death.

b, TBM, tumor-bearing mice.

Total tumor incidence in “effective” control female mice (survived by the time of the death from the first tumor in the experiment) was practically the same in all metformin-treated and control groups (Table 4). Mammary carcinomas and leukaemia developed most frequently, in accord with oncologic characteristics of the female SHR mice [19]. Treatment with metformin failed to influence the total incidence of malignant tumors (Table 4). There were no cases of lung adenocarcinomas in the mice treated with metformin from the age of 3 or 15 months whereas 3 cases of this malignancy were detected in the relevant controls. There was no significant difference in the incidence of any other tumors between mice treated with metformin and controls.

## DISCUSSION

We have found that long-term treatment with the antidiabetic biguanide metformin increased mean life span of female SHR mice when initiated at the young and middle age but not at the old age. Taken together with our previous results [9,10,18-21,24], we conclude that treatment with metformin is most beneficial, in females, when started relatively early in life.

Metformin slowed down disturbances in the estrous function of mice regardless of mice age. Thus, effects of metformin on estrous function can be partially dissociated from extension of lifespan and cancer prevention. Noteworthy, restoration of the hypothalamic sensitivity to estrogens with phenformin restored estrous cycles in 16-month-old female rats [25]. Importantly, metformin improves menstrual regularity, leading to spontaneous ovulation in women with polycystic ovary syndrome [26].

Treatment with metformin did not affect body weight and food consumption as well as metabolic and hormonal parameters, while decreasing body temperature, extending life span and delaying cancer. These data argue against the notion that metformin is a CR mimetic. It was also shown that metformin and CR had different metabolic effects [27-29].

Life span extension by CR may differ among species and depends on the age of onset. Thus, Lipman et al. [30] reported that CR does not extend life span of F344xBNF1 rats when initiated in late middle age (18 months) or in old age (26 months). They had reported similar findings for the Long-Evans rat strain [31]. In contrast, Dhabbi et al. [32] found that CR increases life span of B6C3F1 mice when initiated at 19 months of age. In their turn, Weindruch and Walford [28] reported that CR initiated in 12-month-old mice significantly extended life span, but not as markedly as initiated at weaning. Yu et al. [57] initiated CR in rats at the age of 6 weeks or 6 months. It was found that when CR was limited to the rapid growth period, it did not markedly increase the age of 10th percentile survivors. When CR was initiated after the rapid growth period, it was almost as effective in increasing the age of the 10th percentile survivors as CR initiated at 6 weeks of age [33].

Harrison et al. [34] and Miller et al. [35] studied effect of treatment with rapamycin started at the age of 9 or 20 months. The two data sets were not produced simul-taneously, but they do represent work done using the same conditions of drug preparation, diet, water source, housing, and genetic stocks, with only a 1 year lag between start dates. For male mice, starting rapamycin at 9 months rather than at 20 months did not lead to any improvement in survival. For female mice, there was a suggestion that treatment started at the age of 9 months may lead to some slight decline in mortality risk before 1000 days of age, but comprehensive statistical treatment of the results failed to reveal any significance in the differences [35].

In our study, treatment with metformin did not affect total tumor incidence, but led to the increase in the mean life span of tumor-bearing mice. In contrast, the time of detection of the first tumor was mainly postponed in metformin-treated mice when such treatment has been initiated early and in the middle age.

In transgenic HER-2/neu mice, the similar treatment with metformin did not change the incidence of mammary adenocarcinomas. However, it increased their latency and decreased multiplicity [18]. Antidiabetic biguanides phenformin and buformin inhibited spontaneous and chemically induced carcinogenesis in a number of experimental models [10,11]. Biguanides decreased breast carcinoma risk in diabetes mellitus type 2 [16,18,36,37].

Markers of cellular senescence were studied in fibroblasts obtained from skin of 11-, 16-, 19- and 23-months-old SHR mice treated with metformin since the 3rd and 9th months of life [38]. Significant differences were observed between the average number of senescence-associated heterochromatic foci, the average of area nuclei and fluorescence intensity of nucleus after staining for γ -H2AX in control and metformin-treated animals. Also, it was shown that metformin prevented the accumulation of fibroblasts with large area of nuclei, high activity of senescence-associated β-galactosidase, and high fluorescence intensity after staining for γ -H2AX. Noteworthy, γ -H2AX is also a marker of mTOR-dependent cellular senescence in the absence of DNA damage [39]. Rapamycin decreases levels of γ -H2AX in senescent cells [39]. Short-term dietary restriction reduces levels of γ -H2AX associated with cell senescence in mice [40]. Evidently, metformin delays the “old” cells accumulation and prolongs the organism youth.

Discussing recently the late-life interventions, Flurkey et al. [22] noted that it is unlikely that most people will consider anti-aging treatments when they are young. At the same time, most of the available data show that treatments with various drugs started in younger rodents were more effective in life span extension as compared to these interventions started in late middle or old age [22,34,35,41,42]. Our data are not exception from this rule and suggest “a program of aging” switching on at early age [3,8]. The realization of this program could depend on gender differences as it was shown in our experiments with metformin [21].

## MATERIAL AND METHODS

### Animals

Outbred Swiss-derived female SHR mice were purchased from the “Rappolovo” Animal Farm of the Russian Academy of Medical Sciences. The mice were kept in groups of 5-7 animals in polypropylene cages (30 × 21 × 10 cm) under standard light/dark regimen (12 hours light:12 hours darkness) at 22 ± 2^°^C, and received standard laboratory chow [43] and tap water ad libitum.

### Experimental design

Two hundred and eighty eight female SHR mice were under observations. One hundred and twenty nine mice were intact and served as a control. Other mice were treated with metformin (1, 1-Dimethylbiguanide hydrochloride, Biomedicals, France) with drinking water (100 mg/kg of body weight) daily starting at the age of 3 months (61 mice), 9 months (55 mice) and 15 months (43 mice). The dose of metformin is similar to used in our earlier experiments with SHR, 129/Sv or HER-2/neu mice [10,18-21] and equal to 300 mg/m2 of the surface area. Recalculation for humans gives in average 510 mg/m^2^, that are less than commonly used in clinical practice in diabetics (1.0 - 2.5 g per day). Once a week all mice were palpated for detection of mammary tumors appearance. The localization and the size of tumors were registered on the special charts. Once a month all mice were weighted and, simultaneously, the amount of daily consumed food (g) and water (ml) was measured, and their rates per mouse and per body weight unit were calculated. Every 3 months, vaginal smears of the animals were examined cytologically daily for 2 weeks to estimate the estrous function. In the same period, rectal body temperatures of the mice were measured with an electronic thermometer, TPEM (KMIZ, Russia).

The time of appearance of mammary tumors was evaluated by palpation, and the neoplastic masses were measured with calipers in the two perpendicular diameters. Progressively growing masses of >3 mm in mean diameter were regarded as tumors. At the age of 16.5 months 10 mice from each group were sacrificed by decapitation after overnight fasting. Samples of serum were obtained and stored at the −20^°^C for subsequent metabolic and hormonal analyses. Other animals were observed until their natural deaths. The date of each death was registered, and the mean life span, median, the age at which 90% of the animals died, and the maximum life span were estimated.

### Metabolic and hormonal assays

The serum levels of glucose were estimated by enzyme colorimetric (glucose-oxidase) method with kits from “Impact” (Moscow, Russia); cholesterol and triglycerides - by enzyme colorimetric method with kits of “Olvex” (St.Petersburg, Russia); insulin - by immune enzyme assay (ELISA) with kits from Diagnostic Systems Laboratories, Inc. (U.S.A.).

### Pathomorphological examination

All animals were autopsied. Location, number and size of mammary tumors and their metastases in lungs were checked. All tumors, as well as the tissues and organs with suspected tumor development were excised and fixed in 10% neutral formalin. After the routine histological processing the tissues were embedded into paraffin. 5-7μm thin histological sections were stained with hematoxylin and eosin and examined microscopically. Tumors were classified according to International Agency for Research on Cancer recommendations [44].

### Statistics

Experimental results were statistically processed by the methods of variation statistics with the use of STATGRAPH statistic program kit. The significance of the discrepancies with age-matched controls was defined according to the Student t-criterion, Fischer exact method, χ2, non-parametric Wilcoxon-Mann-Whitney and Friedman RM ANOVA on Ranks. Student-Newman-Keuls Method was used for all pair wise multiple comparisons. Coefficients of correlation were estimated by Spearman method [45]. Differences in tumor incidence were evaluated by the Mantel-Hansel log-rank test.

Parameters of Gompertz model were estimated using maximum likelihood method, non-linear optimization procedure [46] and self-written code in ‘Matlab’; confidence intervals for the parameters were obtained using the bootstrap method [47].

For experimental group Cox regression model [48] was used to estimate relative risk of death and tumor development under the treatment compared to the control group.
